# Black gram Plant Leaf Disease (BPLD) dataset for recognition and classification of diseases using computer-vision algorithms

**DOI:** 10.1016/j.dib.2022.108725

**Published:** 2022-11-04

**Authors:** Srinivas Talasila, Kirti Rawal, Gaurav Sethi, Sanjay MSS, Surya Prakash Reddy M

**Affiliations:** a Lovely Professional University, Phagwara, Punjab, India; b VNR Vignana Jyothi Institute of Engineering and Technology, Hyderabad, India; c Jawaharlal Nehru Krishi Vishwa Vidyalaya, Jabalpur, India; d Malla Reddy University, Hyderabad, India

**Keywords:** Black gram crop (Vigna Mungo), Image datasets, Image classification, Machine Learning, Deep Learning

## Abstract

This article introduces Black gram Plant Leaf Disease (BPLD) dataset, which is scientifically called as *Vigna Mungo* and is popularly known as Urad in India. It is widely considered to be one of the most significant pulse crops farmed in India. Anthracnose, Leaf Crinkle, Powdery Mildew and Yellow Mosaic diseases shown significant impact on the black gram production and causing financial loss to the farmers. A fusion of image processing and computer vison algorithms are widely used in recent years, for applications in the diagnosis and categorization of diseases that affect plant leaves. To detect and classify plant leaf diseases which degrades the quality of the black gram crop, in early stages, using computer vision algorithms, a Black gram Plant Leaf Disease (BPLD) dataset was created and briefly discussed in this article. The dataset holds a total of 1000 images belongs to five classes: four diseases and one healthy. The images in the presented dataset were captured under the real cultivation fields at Nagayalanka, Krishna, Andhra Pradesh, using camera and mobile phones. After the image acquisition, the images were categorized and processed with the help of agriculture experts. Researchers who utilize image processing, machine learning and particularly deep learning algorithms for automated diagnosis and classification of black gram plant leaf diseases in early stage to assist farmers could benefit from this dataset. The dataset is publicly and freely available at https://doi.org/10.17632/zfcv9fmrgv.3

**Specifications Table**

**Table:** 

Subject	Computer Science, Agricultural Science
Specific subject area	Image Processing, Image Classification, Computer Vision, Plant Diseases
Type of data	Raw data, images
How data were acquired	Data acquisition was done with the help of a pathologist and two different devices. The first device is SONY CYBER SHOT DSC-H300 camera with a powerful 35x optical zoom and a resolution of 20.1 Megapixels. The second device is a Samsung Galaxy F41 smartphone:64Megapixels and aperture f/1.89.
Data format	The data are in jpeg format
Parameters for data collection	Disease and healthy leaf images of black gram crop were collected separately. The images that hold the diseases were subcategorized according to their symptoms. The dataset holds Anthracnose, Leaf Crinkle, Powdery Mildew and Yellow Mosaic diseases of black gram crop.
Description of data collection	Data was collected using a camera and smart phone manually and categorized according to the disease characteristics with the help of a pathologist.
Data source location	Nagayalanka, Andhra Pradesh, India PIN:521120.
	Latitude and Longitude of 15.9455° N, 80.9180° E
Data accessibility	Repository Name: Mendeley data
	Data identification number: 10.17632/zfcv9fmrgv.3
	Direct URL to data: https://data.mendeley.com/datasets/zfcv9fmrgv/3
Related Research Article	**Article 1:**
	**Author's Names:** Talasila, S., Rawal, K. and Sethi, G. (2021),
	**Title:** PLRSNet: a semantic segmentation network for segmenting plant leaf region under complex background
	**Journal Name**: International Journal of Intelligent Unmanned Systems
	**DOI:**https://doi.org/10.1108/IJIUS-08-2021-0100.
	**Article 2:**
	**Author's Names:** Talasila, S., Rawal, K., Sethi, G. (2022).
	**Title:** Conventional Data Augmentation Techniques for Plant Disease Detection and Classification Systems
	**Journal Name:** Smart Innovation, Systems and Technologies, Springer
	**DOI:**https://doi.org/10.1007/978-981-19-0011-2_26

## Value of the Data

• The images in dataset contributes visual symptoms of black gram plant leaf diseases such are Anthracnose, Leaf Crinkle, Powdery Mildew and Yellow Mosaic, along with healthy category.
• The dataset enables the researchers to perform computer-vision based approaches for identifying and classifying diseases at their earliest occurrence.
• The dataset helps the researcher to develop computer-vision based algorithms that perform well in the real time scenarios as the dataset was acquired from the real cultivation conditions.
• The dataset is also useful for various Machine Learning or Deep Learning algorithms for the tasks of disease classification, leaf region segmentation, infected region segmentation, disease severity estimation, leaf area estimation and many more.
• The dataset creates a challenging environment for the researchers to identify and classify black gram plant diseases since the images were acquired in real time scenarios.
• Black gram plant leaf disease classification apps can be designed using the presented dataset which can be further used for assisting the farmers for better cultivation of the black gram crop.

## 1 Data Description

A member of pulse tribe, Black gram, also known as Urad bean or Black mapte, is one of the world's most significant and widely consumed pulse. This pulse is in great demand as it has high nutritional content and other advantages like improving soil fertility and fixing atmospheric nitrogen in the soil [1]. India is the primary source with 70% of the world's black gram production. According to 2020-21 reports, India produces around 24.5 lakh tonnes of black gram from approximately 4.6 M hectares of land, with an average of 533 kg/hectare annually. The yield of the crop is reduced significantly because of the most common diseases of black gram crop such are Anthracnose, Leaf Crinkle, Powdery Mildew and Yellow Mosaic. The timely and early recognition and classification of these types of infections is necessary for better crop yield. Considering the above issues, the Black gram Plant Leaf Disease (BPLD) dataset was collected by taking the diseased plant leaf images from the cultivation fields at Nagayalanka, Krishna (d.t), Andhra Pradesh. The dataset has a total of 1000 images of four diseases (Anthracnose, Leaf Crinkle, Powdery Mildew and Yellow Mosaic) and one healthy category. The presented data is partially associated with the article [2]. Table 1 depicts the distribution of images in each disease category. The images were acquired at various times of the day; as a result, they were exposed to a wide range of illumination conditions. The images were captured with the help of SONY CYBER-SHOT DSC-H300 camera and mobile phones. The original RGB images have different dimensions due to the usage of various devices, which were then reduced to 512 × 512 in the preprocessing stage.

**Table 1:** Number of images in each disease category presented in the BPLD Dataset.

Disease of Leaf	Number of images
Anthracnose	230
Healthy	220
Leaf Crinkle	150
Powdery Mildew	180
Yellow Mosaic	220
**Total**	**1000**

The file name contains BPLD dataset, was uploaded in Mendeley data which has five folders in it namely Anthracnose, Healthy, Leaf Crinkle, Powdery Mildew and Yellow Mosaic. The folder name indicates the name of the disease which was present in that particular folder. The first folder namely, Anthracnose contains 230 images of anthracnose disease visual symptoms. Similarly, second folder has 220 images of healthy leaf images, third folder has 150 images of leaf crinkle disease characteristics, fourth folder has 180 images of powdery mildew diseases characteristics and finally fifth folder has 220 images of yellow mosaic disease symptoms. The authors intrusion for collecting the dataset is to design an automated and efficient black gram plant leaf disease classification algorithm using deep learning approach. A detailed description of the disease characteristics is presented below.

### 1.1 Anthracnose - caused by Colletotrichum lindemuthianum (Sexual Stage: Glomerella Lindemuthianum)

**Fig. 1:**
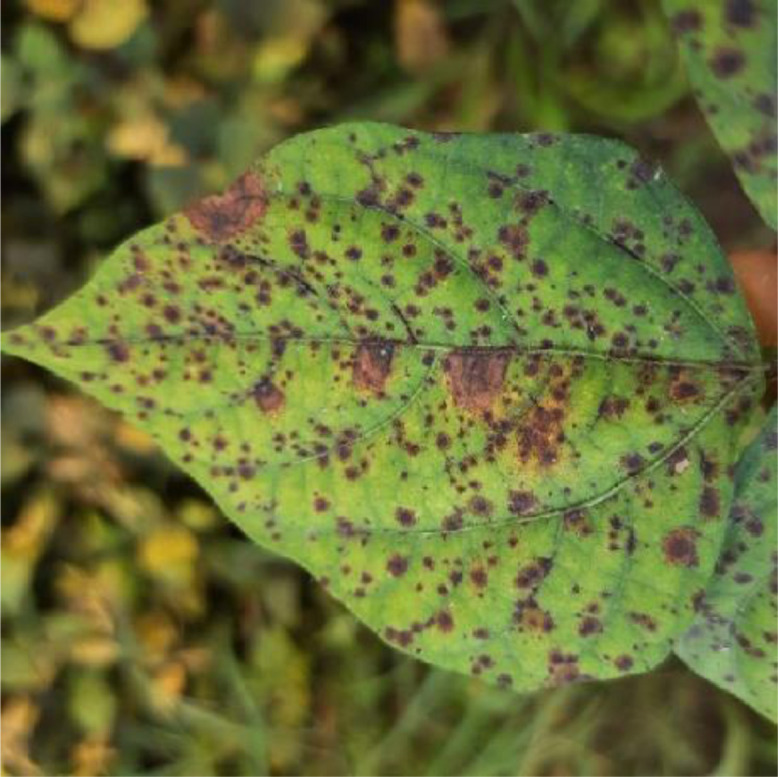
Sample leaf image infected by the Anthracnose disease.

All aerial parts of the plants exhibit symptoms, which may be seen at any phase of the crop's development. On the hypocotyl region, the fungus causes dark brown to black sunken lesions, which eventually cause the seedlings to die. Leaf lesions that are small and angular in shape arise, mainly close to veins, and develop into a greyish white center with a dark brown or reddish edge. Lesions are also be seen on the petioles and stem. At first, tiny water-soaked lesions emerge on the pods, then turn into brown and widen to form a circular, sunken patch with a dark center and bright red or yellow border [3]. The fungus is seed-borne and cause primary infection. Conidia generated on diseased plant parts are perpetuates by the wind and spread as a secondary transmission. A sample leaf image which has been infected by the *Colletotrichum lindemuthianum* is shown in Fig. 1. For this category, 230 images were collected and processed.

### 1.2 Leaf Crinkle - transmitted by Bemisia Tabaci, Weed Hosts Like Aristolochia Bracteata and Digera Arvensi

**Fig. 2:**
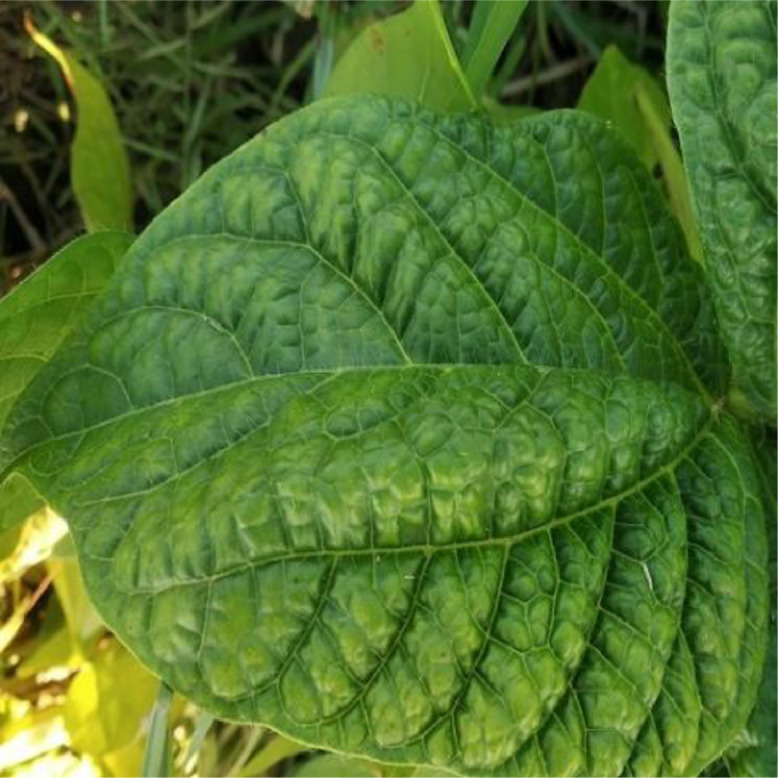
Sample leaf image infected by the Leaf Crinkle disease.

Leaf crinkle disease is a pervasive and catastrophic disease that causes extreme crinkling, puckering, and rugosity in the leaves of black gram plants [4]. The symptoms emerge as chlorosis along certain lateral veins and branches towards the border of the leaf on the youngest leaves. The margins of the leaves seem to be curling downwards. Twisting may also be seen on a few of the leaves. The bottom of the veins is discolored a reddish brown, and also reaches to the petiole. Plants that begin to display symptoms during the first five weeks after planting will inevitably continue to be stunted, with the bulk of these fatalities being caused by top necrosis within a week or two. Plants that have been exposed to the virus during late stages of development do not have extreme curling and twisting of the leaves, but they do display prominent venial chlorosis anyplace on the leaf lamina. The disease spreads mostly by the seed and stroking of infected leaves with the healthy leaves in the field. For this category, 150 leaf crinkle diseased leaf images were captured and utilized for further processing. Fig. 2 below shows the sample leaf image that was infected by the leaf crinkle disease.

### 1.3 Powdery Mildew - caused by Erysiphe Polygoni

**Fig. 3:**
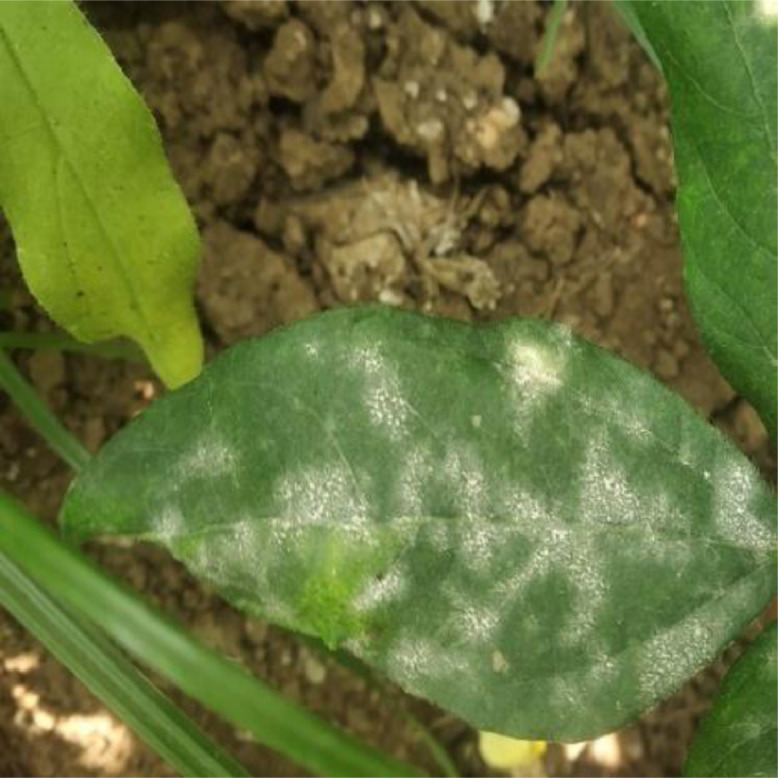
Sample leaf image infected by the Powdery Mildew disease.

Leaves, as well as other green portions are having white powdery patches, which eventually become dull colored. These white patches increases rapidly and cover both the sides of leave circularly. Severely affected parts are distorted and shriveled. The disease also causes infected plants to mature forcibly, causing significant yield losses. The season's conidia, which are carried by the wind, are responsible for secondary spread [5]. *Erysiphe polygoni* is most favorable growth at warm humid weather*.* For this category, 180 powdery mildew diseased leaf images were captured and utilized for further processing. Fig. 3 below represents the sample leaf image that was infected by the powdery mildew disease.

### 1.4 Yellow Mosaic

**Fig. 4:**
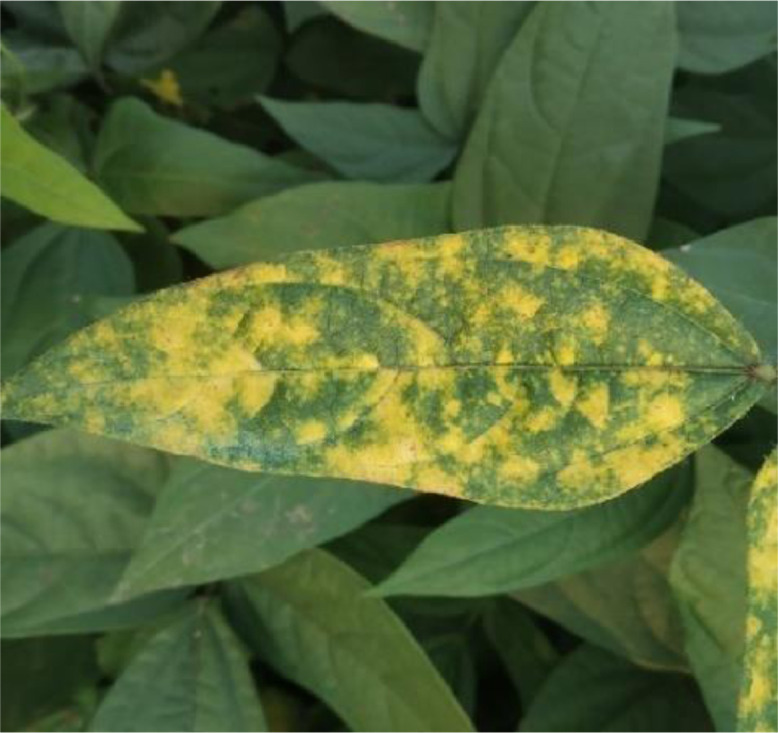
Sample leaf image infected by the yellow mosaic disease.

**Fig. 5:**
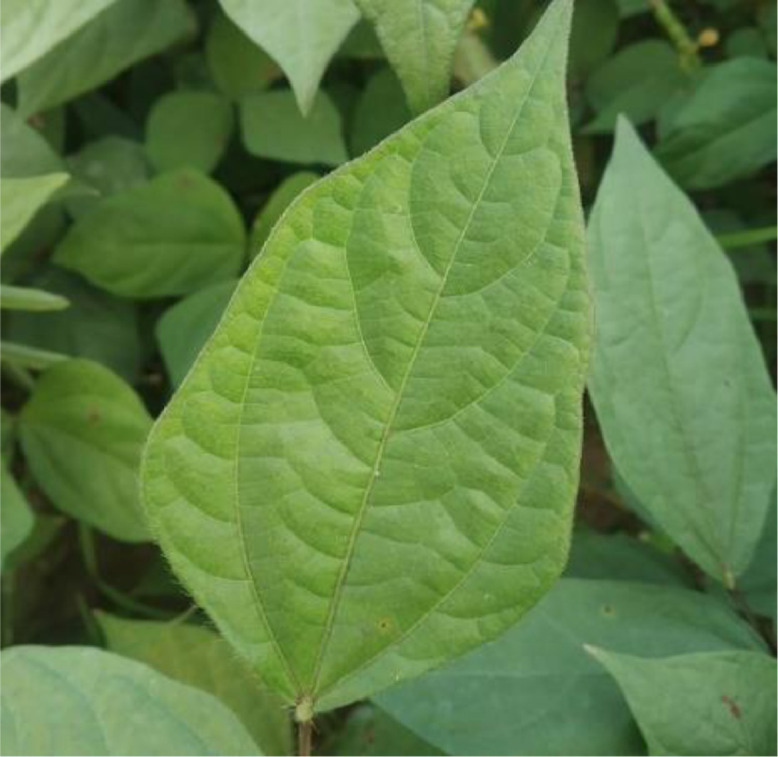
Healthy leave image.

It is the most devastating disease throughout the year, regardless of the season. Crop loss ranges from 50 to 70% as a consequence of this disease, which is particularly severe if it occurs during the early phases of crop development. The first signs of yellowing appear in the form of spots or patches on the young leaves. The yellow discoloration spreads over a larger region, and eventually the whole leaf becomes yellow, dry and withering. A pattern of green and yellow spots appears on infected leaves. Plants infected with the virus mature late. This infection reduces the quality and quantity of the pulse produced by the plants [6]. Whiteflies are the primary transmissions of the virus. For this category, 220 yellow mosaic diseased leaf images were captured and utilized for further processing. Fig. 4 below represents yellow mosaic disease of a black gram plant and Fig. 5 is the healthy leaf image.

## 2 Experimental Design, Materials and Methods

**Fig. 6:**
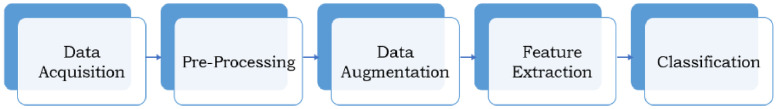
Steps involved in implementing disease detection and classification algorithms.

Implementation of plant disease detection and classification algorithms must go through four stages before they can be put into action. Fig. 6 shows the four key stages of classification algorithms.

### 2.1 Data acquisition

At this stage, high-resolution digital cameras/mobile phones might be used to capture diseased leaf images from the fields [7], [8], [9]. In this work, SONY CYBER-SHOT DSC-H300 camera and Samsung mobile phone were utilized to capture the images in the BPLD dataset. Later, all the images in the dataset were categorized with the help of a pathologist who supported with the description of the diseases. The coastal Andhra region in Andhra Pradesh is well known for black gram production. So, few visits to Nagayalanka Krishna (D.T), Andhra Pradesh were made between the months of Jan-April 2020 & 2021, where the black gram is cultivated in that specific period of time. As described by the pathologist, identified & captured most common diseases of black gram crop such are Anthracnose, Leaf Crinkle, Powdery Mildew and Yellow Mosaic. A total of 1000 images were collected, and details of each disease category is presented in Section 1.

### 2.2 Pre-Processing

The original RGB images had different dimensions due to the usage of various devices. Each image in the dataset was then scrutinized for the square dimension. Later, the images that were not in square, a cropping tool was used to get the whole leaf region with squared dimensions. Finally, all squared dimension images were rescaled to 512 × 512 for further processing.

### 2.3 Data Augmentation

**Fig. 7:**
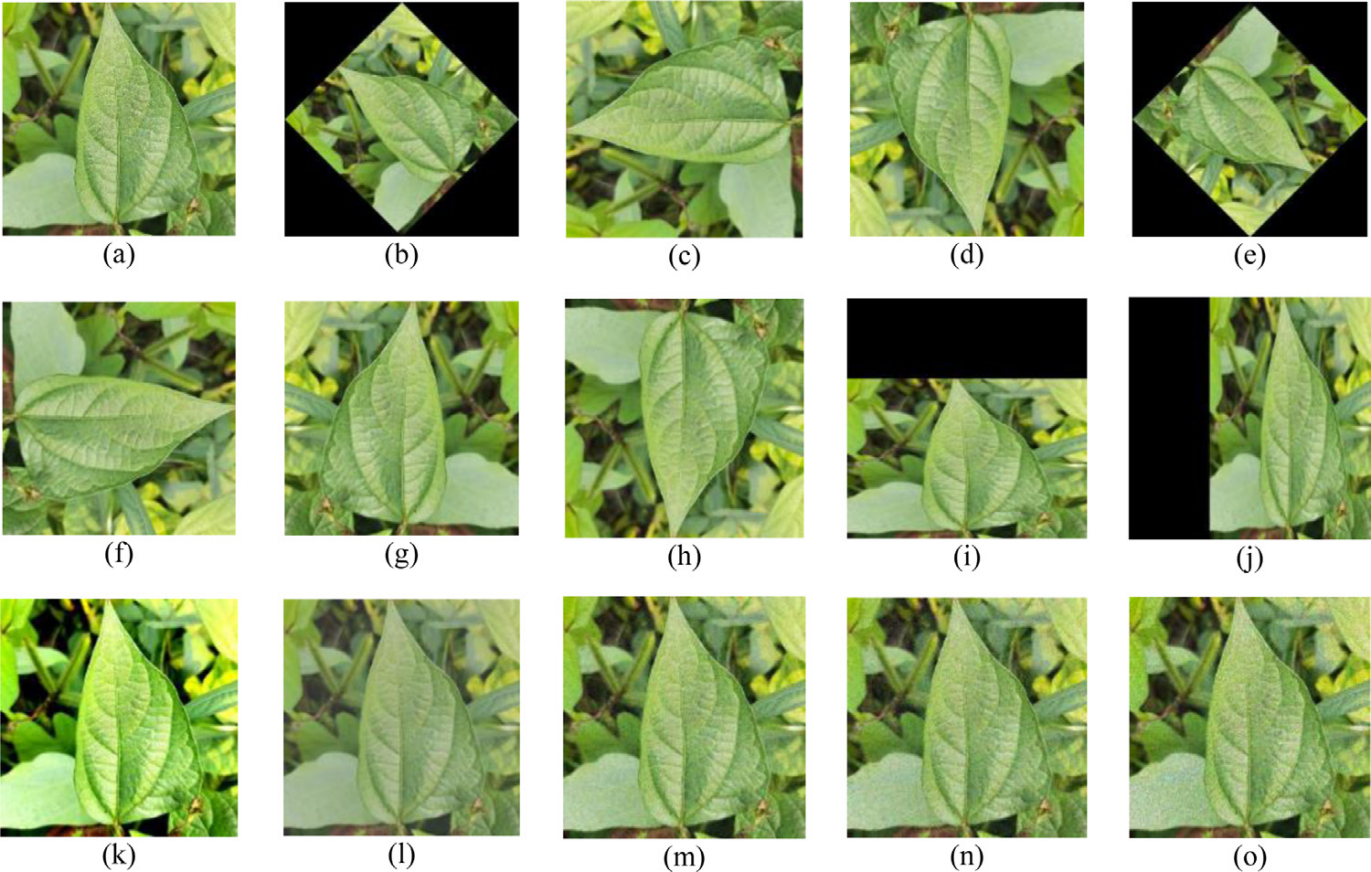
Data augmentation techniques (a) Original image (b) 45° rotation (c) 90° rotation (d) 180° rotation (e) 225° rotation (f) 270° rotation (g) vertical symmetry (h) horizontal symmetry (i) shifting vertically by 256 pixels (j) shifting horizontally by 256 pixels, (k) & (l) adjusting various image intensity values (m) gaussian noise (n) salt & pepper noise (o) speckle noise.

The effectiveness of classification models designed to recognize and categorize plant diseases is contingent on the availability of a substantial quantity and diversity of the data. The acquisition of such a large quantity of data, however, is reliant on various factors such as atmospheric conditions, change of sunlight, absence of disease at that particular time etc. In order to solve these issues data augmentation techniques are going to be quite helpful. The term ‘data augmentation’ refers to a method of producing a large amount of data from limited amount of accessible data. The basic data augmentation techniques or deep learning-based data augmentation techniques can be used to enhance the dataset based on the requirement. Data augmentation techniques helps to improve the designed model's performance and to reduce overfitting problem, which is crucial in deep learning. In this work, 15 data augmentation techniques were employed, such are belonging to rotation, mirror symmetry, illumination correction, shifting/translation, and noise injection [10]. Fig. 7 below depicts various data augmentation techniques that were utilized to increase the dataset.

### 2.4 Feature Extraction and Classification

Machine learning or deep learning models may be used to perform feature extraction and classification for effectively identifying and classifying plant leaf diseases. In machine learning, feature extraction is an essential phase in constructing any pattern recognition and attempts to obtain the appropriate information that classifies each class [11]. Feature extraction aims at extracting most important information from the raw images. The extraction of features from advanced machine learning techniques is implemented in a learning algorithm that automatically extracts features without any interference by a human expert. The image features include Correlation, Entropy, Variance, Homogeneity, Contrast, Energy, and Mean are computed. The classifiers then associate the input with the target output by using these image features. In deep learning, both feature extraction and classification tasks are performed by Convolutional Neural Networks (CNNs). Because CNNs does not require any manually extracted features since they can extract features by themselves. As an example of the deep learning models for classifying black gram plant leaf diseases mentioned in this article, we trained a few CNN models using the transfer learning technique on the dataset presented with the aforementioned augmentation techniques.

**Fig. 8:**
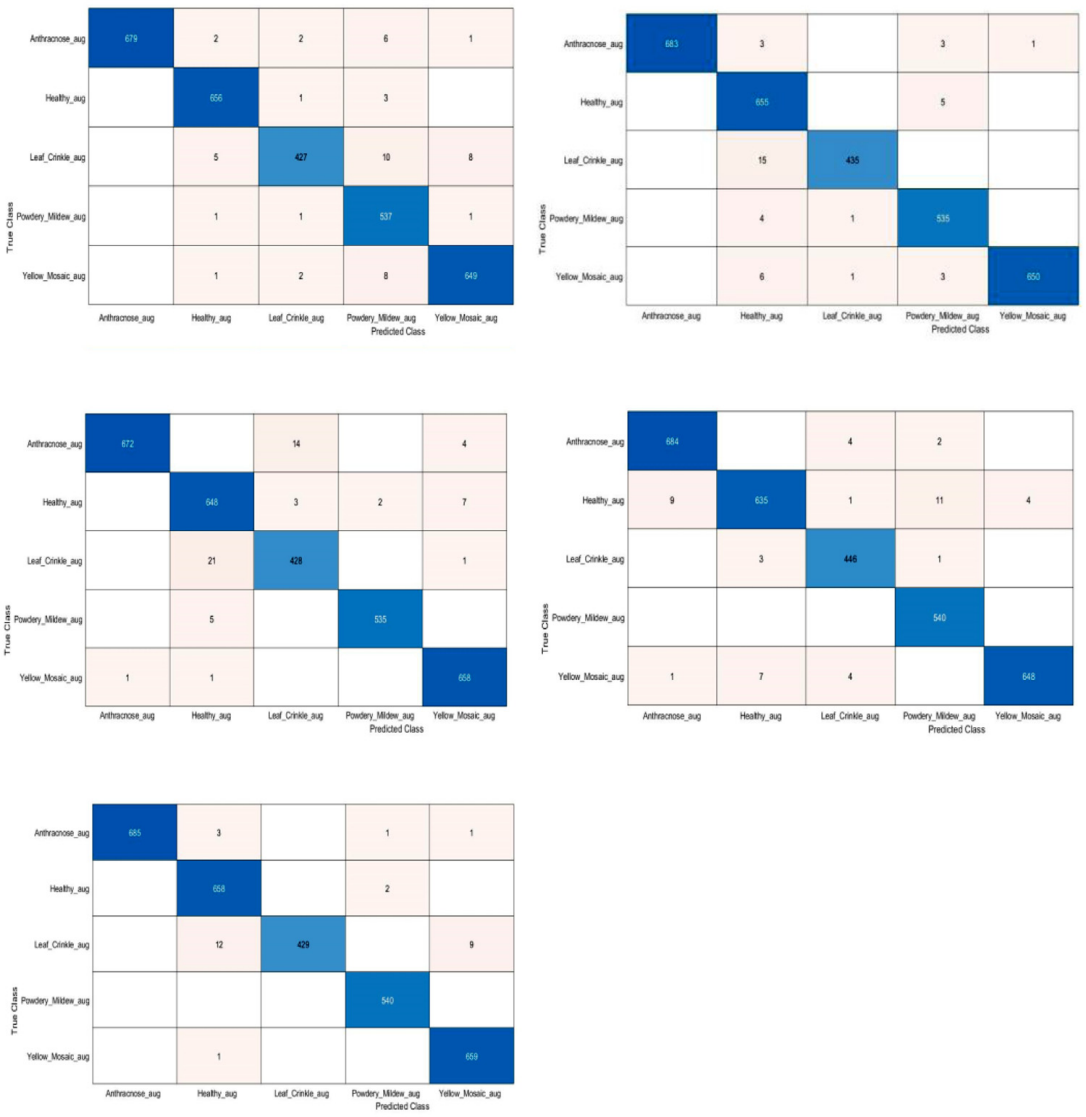
Confusion metrices of the ResNet18 using 5-fold cross-validation technique.

Initially, after the preprocessing, the dataset was enhanced to 15000 images by employing data augmentation techniques presented in section 2.3. K-fold cross-validation was utilized to train the models for measuring the capability of the model's performance on an unseen data. We have set K value as 5 so that the dataset was divided into 5 folds. Therefore, each fold contains 3000 images of all diseased categories. The hyperparameters such as SGDM solver, 0.001 initial learning rate, 32 mini batch size and 30 epochs were used to train all the adopted models. The average performance values of experiments using 5-fold cross-validation were presented in Table 2. The experimental results revealed that the residual network (ResNet-18) with an 18-layer depth outperformed the other trained models with a precision of 98.46%, recall of 98.37%, F1_Score of 98.40% and average classification accuracy of 99.39%. Fig. 8 illustrates the confusion metrices of ResNet-18 using 5-fold cross validation technique.

**Table 2:** Average performance values of the adopted CNN models using 5-fold cross-validation technique.

Model	Input Size	Precision	Recall	F1 Score	Accuracy
AlexNet	227 × 227	93.94%	92.73%	93.05%	97.39%
GoogLeNet	224 × 224	97.70%	96.98%	97.25%	98.97%
MobileNetV2	224 × 224	98.07%	97.86%	97.95%	99.21%
ResNet18	224 × 224	98.46%	98.37%	98.40%	99.39%
EfficientNetB0	224 × 224	97.67%	97.12%	97.32%	98.96%

## Ethical Statement

There are no animal studies by any of the authors in this article

## CRediT Author Statement

**Srinivas Talasila:** Investigation of data collection methods, Methodology, Conceptualization; **Dr. Kirti Rawal:** Writing – original draft preparation; **Dr. Gaurva Sethi:** Writing – review & editing; **Sanjay MSS** and **Surya Prakash Reddy M:** Data Verification

## Declaration of Competing Interest

The authors declare no competing financial or personal of interests.

## Data Availability

Blackgram Plant Leaf Disease Dataset (Original data) (Mendeley Data).
